# Cross-genotype protection of live-attenuated vaccine candidate for severe fever with thrombocytopenia syndrome virus in a ferret model

**DOI:** 10.1073/pnas.1914704116

**Published:** 2019-12-09

**Authors:** Kwang-Min Yu, Su-Jin Park, Min-Ah Yu, Young-Il Kim, Younho Choi, Jae U. Jung, Benjamin Brennan, Young Ki Choi

**Affiliations:** ^a^Department of Microbiology, College of Medicine and Medical Research Institute, Chungbuk National University, Cheongju 28644, Republic of Korea;; ^b^Zoonotic Infectious Diseases Research Center, Chungbuk National University, Cheongju 28644, Republic of Korea;; ^c^Department of Molecular Microbiology and Immunology, Keck School of Medicine, University of Southern California, Los Angeles, CA 90033;; ^d^Medical Research Council–University of Glasgow Centre for Virus Research, Institute of Infection, Immunity and Inflammation, College of Medical, Veterinary and Life Sciences, University of Glasgow, Glasgow G61 1QH, Scotland, United Kingdom

**Keywords:** SFTS, emerging banyangvirus, live attenuated vaccine, ferret model, bunyavirus

## Abstract

Severe fever with thrombocytopenia syndrome (SFTS) virus (SFTSV) is an emerging tick-borne virus classified within the *Banyangvirus* genus. SFTS disease has been reported throughout East Asia since 2009 and is characterized by high fever, thrombocytopenia, and leukopenia and has a 12 to 30% case fatality rate. Due to the recent emergence of SFTSV, there has been little time to conduct research into preventative measures aimed at combatting the virus. SFTSV is listed as one of the World Health Organization’s Prioritized Pathogens for research into antiviral therapeutics and vaccine development. Here, we report 2 attenuated recombinant SFTS viruses that induce a humoral immune response in immunized ferrets and confer complete cross-genotype protection to lethal challenge. Animals infected with rHB29NSsP_102_A or rHB2912aaNSs (both genotype D) had a reduced viral load in both serum and tissues and presented without high fever, thrombocytopenia, or mortality associated with infection. rHB29NSsP_102_A- or rHB2912aaNSs-immunized animals developed a robust anti-SFTSV immune response against cross-genotype isolates of SFTSV. This immune response was capable of neutralizing live virus in a focus-reduction neutralization test (FRNT) and was 100% protective against a cross-genotype lethal challenge with the CB1/2014 strain of SFTSV (genotype B). Thus, using our midsized, aged ferret infection model, we demonstrate 2 live attenuated vaccine candidates against the emerging pathogen SFTSV.

## Significance

Severe fever with thrombocytopenia syndrome virus (SFTSV) is an emerging viral pathogen discovered in 2009. The virus is present in countries of East Asia and is transmitted through the bite of an infected *Haemaphysalis longicornis* tick. SFTSV disease is associated with high morbidity and is often fatal. Despite the incidence of disease, no antiviral therapy or vaccine has been approved for use. Here, we report and assess 2 live attenuated viruses as vaccine candidates in our recently described ferret model of infection. We show that the viruses caused no clinical disease or mortality in healthy animals. Immunized animals mounted a robust humoral immune response to a single dose of virus, and this response protected the animals from a lethal challenge.

Severe fever with thrombocytopenia syndrome (SFTS) virus (SFTSV) is an emerging viral pathogen classified within the *Huaiyangshan banyangvirus* species, *Banyangvirus* genus of the *Phenuiviridae* family (1). First reported in China in 2009 (2), cases of SFTS disease and subsequent virus isolations have been described in Japan (3), South Korea (4–6), and more recently in Vietnam (7). The virus is considered by the World Health Organization to be a pathogen “likely to cause wide epidemics” and requires urgent scientific attention to be directed toward the development of antiviral therapies and novel vaccines (8, 9).

SFTSV is maintained in nature by an enzootic tick–suspected animal–tick cycle (10). *Haemaphysalis longicornis* is implicated as one of the main vectors of SFTSV (11) and their increased activity from March through November correlates with the epidemic season of SFTSV (10, 12). SFTSV infection in humans is believed to be predominantly mediated through the bite of a virus-infected tick. However, in 2012, human-to-human transmission of SFTSV was described through contact with, or exposure to, blood of SFTS index patients (13). Subsequent reported human-to-human transmissions of SFTSV are thought to have occurred among families, residents of villages where patients lived, and even in hospital settings (14–16). When secondary SFTS cases occurred, they were often fatal and displayed similar symptoms of infection to those seen in the index patients, presenting with high viral loads in serum and low platelet counts being reported (17).

SFTSV causes an often-fatal disease (12 to 15% case fatality rate) that is characterized by thrombocytopenia, hemorrhagic manifestations, and multiorgan failure (2, 18). To date, there have been ∼8,500 reported cases of SFTS disease in China, more than 100 laboratory confirmed cases in both South Korea (4–6) and Japan (3, 19), and 2 cases reported in Vietnam (7).

Members of the *Phenuiviridae* family contain a trisegmented single-stranded RNA genome of negative or ambisense polarity, encoding 5 or 6 proteins (20, 21). The 3 genomic RNA segments are designated the large (L), medium (M), and small (S) segments. The L segment encodes the viral RNA-dependent RNA polymerase, the M segment encodes the 2 viral envelope glycoproteins (Gn and Gc), and the S segment encodes the nucleocapsid protein (N) and a nonstructural protein (NSs) in an ambisense manner (22).

Phylogenetic analysis of SFTSV strains isolated to date suggest that 6 genotypes (A to F) circulate in East Asia, and that genotype reassortment is commonly detected. The genotyping roughly correlates phylogeographically, although the nomenclature in the current literature is not consistently applied. Genotypes A, B, D, and F have been isolated in South Korea, genotypes B and E in Japan, and all genotypes have been isolated in China (12, 23, 24).

The nonstructural protein (NSs) of phenuiviruses, in particular that of SFTSV, is a well-characterized innate immune antagonist and virulence factor (25–31). NSs acts as an antagonist to IFN signaling to evade the expression of antiviral genes (28, 31, 32). Previously, we have described 2 viruses that are unable to interact with mammalian innate immune pathways such as the IFN-β induction and signaling cascades and the TPL2 signaling pathway. These viruses, rHB2912aaNSs (33) and SFTSV-PA (herein known as rHB29NSsP_102_A) (31), contain a carboxyl-terminal truncated short 12 amino acid peptide sequence derived from the NSs open-reading frame (ORF) or have a single point mutation in the NSs coding sequence from proline to alanine at position 102, respectively. Both viruses demonstrated an inability to antagonize mammalian innate immune responses to viral infection in vitro. Additionally, rHB29NSsP_102_A was shown to be attenuated in vivo in C57BL/6 wild-type mice that had been pretreated with an anti-IFN-α/β receptor 1 (IFNAR1)-blocking antibody prior to infection (31).

Many rodent models for SFTSV have been developed in recent years, but no one model completely recapitulated the SFTS disease phenotype seen in human cases (34). Infection with SFTSV is only lethal to newborn rodents or genetically modified immunocompromised animals (35–38); even experimentally infected Rhesus macaques (39) and Cynomolgus macaques (34) do not develop a lethal disease. Recently, we described an immunocompetent age-dependent ferret model for SFTSV that recapitulated all SFTS disease pathologies, as evidenced by high viral loads in serum and tissues, thrombocytopenia, leukopenia, and a 93% mortality rate (40).

Since the discovery of the virus, there has been minimal work published on the development of antiviral therapies or vaccine candidates, and there is currently no commercially available vaccine to prevent SFTSV. Thus, it is of high priority to develop and evaluate potential vaccines to control and halt the spread of this rapidly emerging infectious agent. Work has focused on a recombinant NSs protein vaccine (41), a DNA vaccine based on plasmids expressing the N or NSs proteins (42) or a recombinant vesicular stomatitis virus (rVSV)-based vaccine candidate expressing the SFTSV Gn/Gc glycoproteins (43). One therapeutic agent has also been developed through the characterization of an antibody isolated from an infected patient. Here, the authors demonstrated that this antibody conferred protection to a lethal SFTSV challenge (44).

In this paper, we bring together previously developed recombinant viruses and the newly described ferret model of infection to assess the efficacy of these viruses as live attenuated vaccine candidates. We show that while infection with a genotype D Korean strain of SFTSV (CB8/2016) was lethal in our ferret model, infection of the animals with recombinant viruses, including a wild-type derived recombinant Chinese isolate (rHB29; genotype D) resulted in a mild self-resolving infection devoid of fever. Infected animals experienced no thrombocytopenia, had reduced viral RNA levels in both serum and tissues and all animals survived infection. These animals developed a robust anti-SFTSV humoral immune response against homologous and heterologous genotypes of SFTSV by 14 d post inoculation and this immune response was 100% protective against a cross-genotype lethal challenge with the CB1/2014 strain (genotype B). Further, rHB2912aaNSs showed no reversion to wild type during serial passage in vitro and in vivo. It is of note, that both CB8/2016 and HB29 are genotype D wild-type viruses isolated from infected patients. However, as demonstrated, HB29 is less virulent in our ferret model of infection. The further deletion or mutation of the NSs ORF as seen in rHB2912aaNSs or rHB29NSsP_102_A respectively, further attenuates these viruses (2, 45).

Thus, we demonstrate genetically stable, safe, live attenuated vaccine candidates against the emerging pathogen SFTSV.

## Results

### Recombinant SFTS Viruses Do Not Induce Lethal Disease in a Ferret Model.

We recently described an age-dependent ferret model for SFTS disease where infected animals demonstrated reduced white blood cell counts, severe thrombocytopenia, high fever and a high mortality rate (40). We used this model to assess the virulence of recently described recombinant SFTS viruses (rHB2912aaNSs and rHB29NSsP_102_A) and assessed their suitability as live-attenuated vaccine candidates (31, 33).

**Fig. 1.**
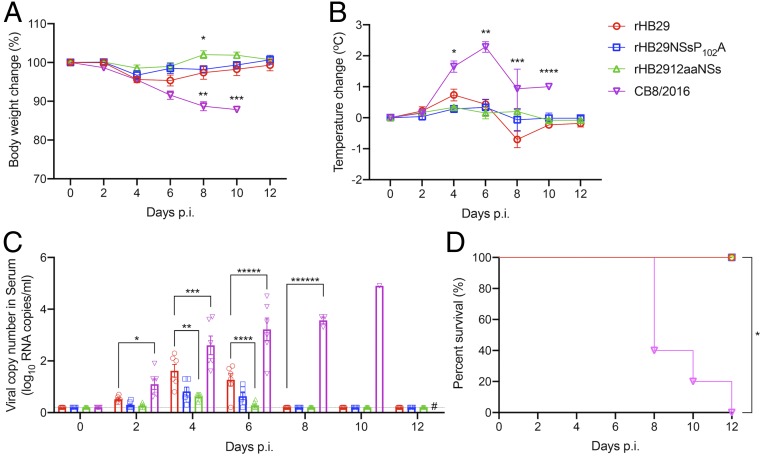
Relative body weight, temperature change, serum viral RNA copy number, and survival of aged ferrets infected with recombinant SFTS viruses. (*A*–*D*) Twelve ferrets in each group were inoculated with 4 × 10^6^ PFUs of rHB29 (red), 4 × 10^6^ PFUs of rHB29NSsP_102_A (blue), 5 × 10^5^ PFUs of rHB2912aaNSs (green), or 5 × 10^5^ PFUs of CB8/2016 (purple). Relative weight (*A*), temperature (*B*), viral RNA copy number in the serum (*C*), and survival (*D*) were assessed at the indicated times p.i., and mean values are shown ± SEM. The asterisks indicate significance compared to each day p.i. sample analyzed by one-way ANOVA with Dunnett multiple comparison test (*A*–*C*) or the 2-tailed Mantel–Cox method (*D*). *P = 0.0424, **P = 0.0023 and ***P < 0.0134 (*A*); *P = 0.0007, **P < 0.0001, ***P = 0.0113 and ****P = 0.0032 (*B*); *P = 0.0031, **P = 0.0192, ***P = 0.0214, ****P = 0.0378, *****P = 0.0001, and ******P < 0.0001, (*C*); and *P < 0.0001 (*D*). # Sample not tested as animals had died/been killed.

Groups of 12 ferrets were inoculated intramuscularly (i.m.) with 2 wild-type viruses: The first, a Korean strain of SFTSV (CB8/2016; *SI Appendix*, Table S1); the second, recombinant mutant SFTS viruses from genotype D that were isolated in China (all based on sequences from a Chinese patient isolate, HB29; *SI Appendix*, Table S1). Body temperature and weight-change measurements were recorded and blood collection for hematology analysis were performed on 0-, 2-, 4-, 6-, 8-, 10-, and 12-d postinfection (p.i.). The results showed that although rHB29-infected animals showed slightly increased fever and weight loss over time, significant weight loss and fever were not observed in animals infected with rHB29 or either of the NSs-mutant viruses. Animals infected with the CB8/2016 strain recorded an average weight loss of 12% by 8 d p.i. (Fig. 1*A*), and a marked fever was observed from 4 d p.i. (2.3 °C increase). The recorded fever remained high until the cessation of the experiment (Fig. 1 *A* and *B*).

To demonstrate the association between clinical symptoms and virus replication in infected animals, we tested for the presence of viral RNA in the serum by qRT-PCR. All 4 viruses were detected in the sera of infected animals with rHB29 replicating to statistically higher levels than rHB2912aaNSs at days 4 and 6 p.i., whereas viral RNA from CB8/2016 was detected in the sera of infected animals from day 2 p.i. Peak RNA amounts of 1.62, 0.82, and 0.62 log_10_ viral copies per 1 mL were observed at 4 d p.i. in rHB29-, rHB29NSsP_102_A-, and rHB2912aaNSs-infected animals, respectively, before being cleared to undetectable levels by day 8 p.i. However, in animals infected with strain CB8/2016, viral RNA levels rose consistently over the course of the experiment peaking at 4.9 log_10_ viral copies per 1 mL at 10 d p.i. Viral copy numbers of CB8/2016 recorded in the serum were consistently and statistically greater than those recorded in animals infected with the other SFTS viruses (Fig. 1*C*). Three of the animals infected with CB8/2016 died at day 8 p.i., and all other ferrets infected with CB8/2016 succumbed to infection by 12 d p.i. No mortality was associated with infection of animals with either rHB29, rHB29NSsP_102_A, or rHB2912aaNSs viruses (Fig. 1*D*).

### Tissue Distribution of Recombinant SFTS Viruses.

**Fig. 2.**
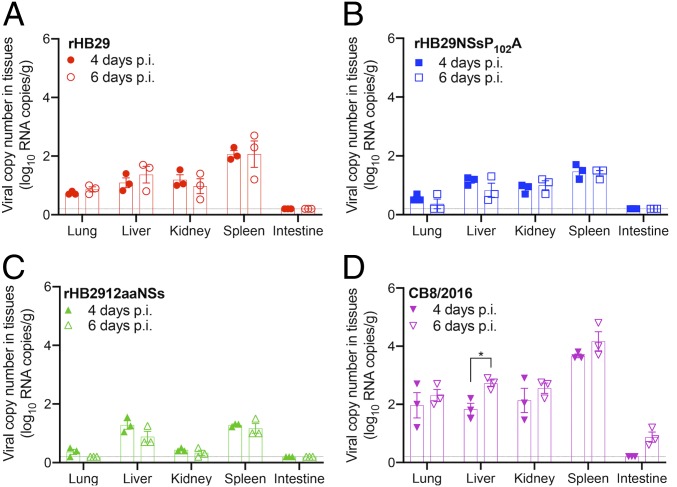
Distribution and viral RNA copy number in tissues of SFTSV-infected ferrets. Tissues (*n* = 3 per group) from ferrets infected with rHB29 (*A*), rHB29NSsP_102_A (*B*), rHB2912aaNSs (*C*), or CB8/2016 (*D*) were collected at days 4 (solid shapes) and 6 p.i. (open shapes), and viral RNA copy numbers were assessed with real-time PCR. Examined tissues included lung, liver, kidney, spleen, and intestine. The asterisks indicate significance compared to each day p.i. sample by the 2-tailed, unpaired *t* test (*P = 0.0195). Each experiment was performed for 3 biological and 3 technical repeats.

To further demonstrate the dissemination of rHB29, rHB29NSsP_102_A, and rHB2912aaNSs in infected ferrets, 3 ferrets of each group were killed at days 4 and 6 p.i., and viral RNA copy numbers in lung, liver, kidney, spleen, and intestines were assessed by qRT-PCR (Fig. 2). In rHB29-infected animals, viral RNA was detectable at low levels in all tissues with the exception of intestine. Peak virus copy number (2.1 log_10_ viral copies/g) was recorded in the spleen at day 6 p.i. (Fig. 2*A*). This pattern of tissue tropism and replication was also observed in the tissue samples assayed from rHB29NSsP_102_A-infected animals (Fig. 2*B*). This result was expected as these viruses only differ by one amino acid within the NSs ORF (31). When tissues from ferrets infected with rHB2912aaNSs were analyzed, a difference in virus tissue distribution was noted. rHB2912aaNSs was only detected in the liver and spleen of infected animals (1.29 log_10_ viral copies/g), peaking at 4 d p.i. Viral genome copy numbers detected in the lung, kidney, and intestines of these animals were recorded at or just above the limit of detection (Fig. 2*C*).

In CB8/2016-infected animals, viral RNA levels were high in all tissues assayed, with peak viral RNA levels detected in the spleen (4.2 log_10_ viral copies/g) at 6 d p.i. A statistically significant increase in CB8/2016 virus copy number from 68 to 525 genome copies was also noted in the liver between 4 and 6 d p.i., respectively. CB8 replication was also detected in the intestines of infected ferrets (0.87 log_10_ viral copies/g) at day 6 p.i., demonstrating a higher virulence and systemic spread of this virus compared to the other 3 viruses tested (Fig. 2*D*).

### Hematological Analysis of Blood from Infected Ferrets.

**Fig. 3.**
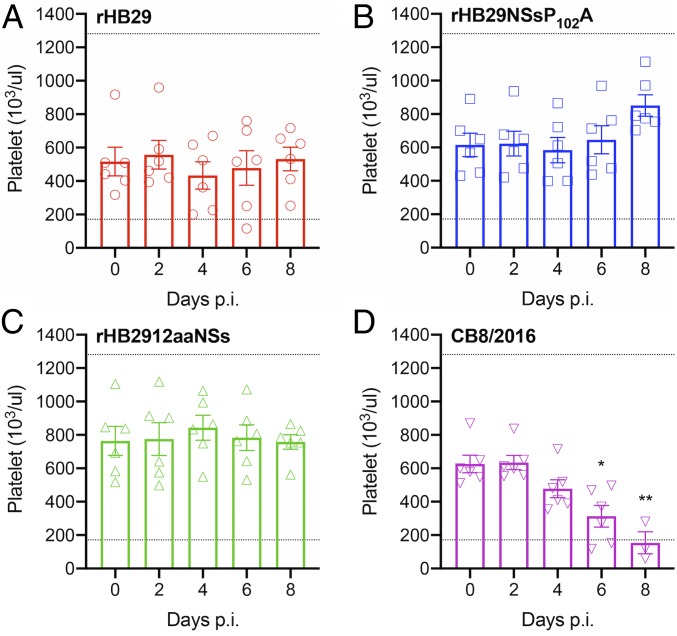
Hematological analysis of platelets in SFTSV-inoculated ferrets. Blood from infected ferrets was collected every other day, and hematological examination was performed using a Celltac hematology analyzer. Platelet counts from rHB29 (*A*), rHB29NSsP_102_A (*B*), rHB2912aaNSs (*C*), or CB8/2016 (*D*) are shown. Normal ranges of platelet counts in ferrets are 171.7 to 1,280.6 × 10^3^/µL and 2.5 to 16.7 × 10^6^/µL, respectively (46, 58). The dashed lines indicate the normal values of platelet counts. The asterisks indicate significance compared to each day p.i. sample by one-way ANOVA with Dunnett multiple comparison test. *P = 0.0404 (*A*) and *P = 0.0016 and **P = 0.0002 (*D*). Each experiment was performed for 3 biological and 3 technical repeats.

One of the hematological pathologies’ characteristic of SFTS disease is a marked thrombocytopenia in infected patients and animals (2, 40). We examined platelet levels in the blood of animals infected with the 4 different viruses (Fig. 3). The expected normal range of platelets in laboratory ferrets is 171.1 to 1,280.6 × 10^3^/μL (46). From 4 d p.i., several animals infected with rHB29 recorded platelet counts lower than the paired control sample taken on day 0 p.i. However, there was no statistical reduction in mean platelet counts observed at any time point analyzed (Fig. 3*A*). None of the animals infected with either of the NSs-mutant viruses developed thrombocytopenia over the course of infection, and recorded mean platelet counts in these groups remained steady or even increased with time (Fig. 3 *B* and *C*). CB8/2016-infected animals developed a statistically significant thrombocytopenia by 6 d p.i., and this continued until 8 d p.i., where several animals had platelet counts below the expected normal range (Fig. 3*D*). The average platelet count at 8 d p.i. for animals infected with CB8/2016 was only 153 × 10^3^/μL, compared to that of 533, 851, or 758 × 10^3^/μL in blood from rHB29-, rHB29NSsP_102_A-, or rHB2912aaNSs-infected animals, respectively.

Hematological cell counts and blood chemistries were also analyzed on blood sampled from the infected animals (*SI Appendix*, Fig. S1). A decrease in white blood cell count was only observed in CB8/2016-infected ferrets at 6 d p.i. (*SI Appendix*, Fig. S1*A*). No decrease in red blood cell counts were observed (*SI Appendix*, Fig. S1*B*). Alanine aminotransferase (ALT) or aspartate (AST) levels were also measured in the infected blood samples. Increased ALT and AST levels were detected in rHB29- and CB8/2016-infected samples over time, whereas no changes were detected in either enzyme level following rHB2912aaNSs infection (*SI Appendix*, Fig. S1 *C* and *D*).

### Infection with Recombinant SFTS Viruses Induce Robust Antibody Responses in SFTSV-Infected Animals.

**Fig. 4.**
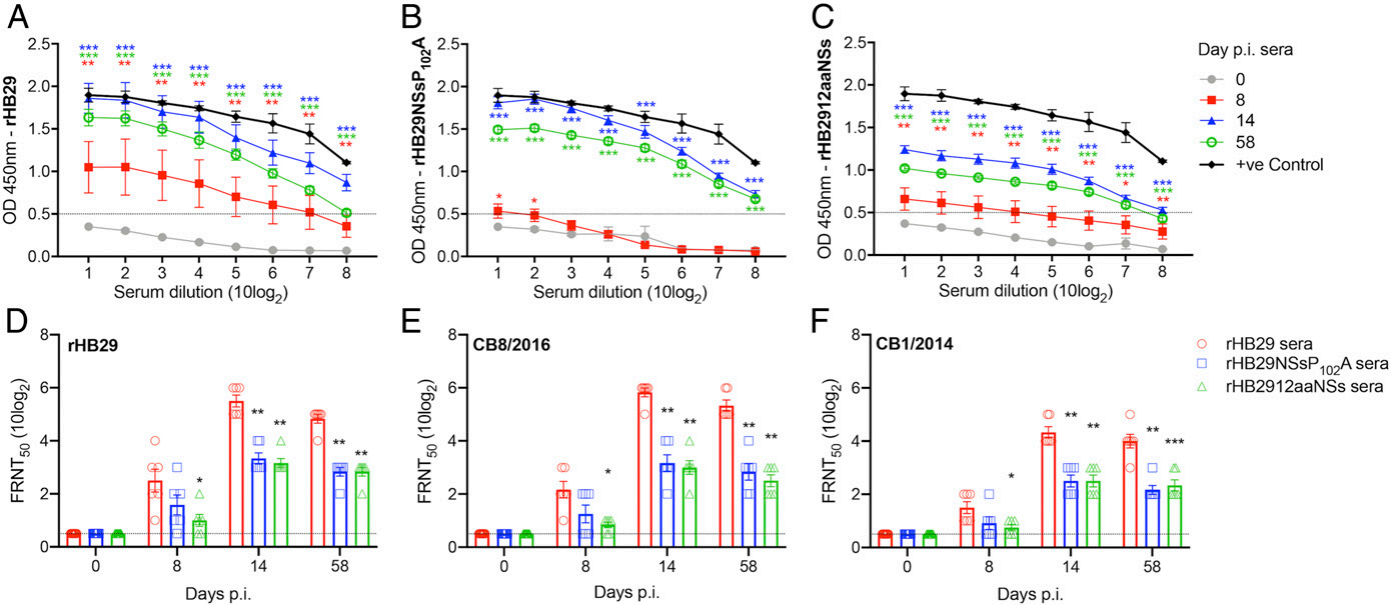
Specific humoral immune responses detected in infected ferrets. Serum from ferrets surviving an initial infection with rHB29 (*A*), rHB29NSsP_102_A (*B*), or rHB2912aaNSs (*C*) were analyzed at 0 (gray), 8 (red), 14 (blue) and 58 (green) days p.i. Positive ferret serum (black) was added as a control for comparison. Humoral immune responses were analyzed using a nucleocapsid protein (N)-based ELISA. Optical density (OD) was measured with a spectrometer (iMark Microplate Reader; Bio-Rad) at 450 nm. The cutoff value was set at an OD value of 0.5 and is indicated as a dashed line in A–C. Serum neutralization assays with rHB29 (*D*), CB8/2016 (*E*), and CB1/2014 (*F*) virus strains were analyzed by FRNT_50_ at 0, 8, 14, and 58 d p.i. The asterisks indicate significance compared to the respective serially diluted day 0 p.i. sample (*A*–*C*) or to the rHB29-immunized serum test (*D*–*F*), assessed by one-way ANOVA with Dunnett multiple comparison test. *P < 0.05, **P < 0.01, or *** < 0.0001 (*A*–*C*); *P = 0.0165 and **P < 0.0001 (*D*); *P = 0.0061, **P < 0.0001, (*E*); and *P = 0.0328, **P < 0.0001, and ***P = 0.0001 (*F*).

To investigate the immunogenicity of the recombinant viruses, we examined the anti-SFTSV humoral immune responses in infected ferrets. A nucleocapsid protein (N)-based enzyme-linked immunosorbent assay (ELISA) was performed with serially diluted ferret sera collected at 0, 8, 14, and 58 d p.i. (Fig. 4 *A*–*C*). Sera from rHB29-infected ferrets showed an increase in optical density (OD) values measured at day 8 and day 14 p.i.. It is noteworthy that although the OD values recorded in sera from rHB29NSsP_102_A- and rHB2912aaNSs-infected ferrets at 8 d p.i. were lower than that of parental rHB29-infected group, both groups showed significantly increased OD values (compared to day 0 p.i.) by 14 d p.i. The antibody responses seen may be correlated with the relatively low virus RNA detected in sera and tissues of rHB29NSsP_102_A- or rHB2912aaNSs-infected ferrets. At 14 d p.i., OD values of 1.0 were achieved with dilutions of 7.63, 7.10, or 4.11 (10log_2_) of rHB29-infected (Fig. 4*A*), rHB29P102A-infected (Fig. 4*B*), or rHB2912aaNSs-infected (Fig. 4*C*) animal sera, respectively. These data demonstrate that a reduced humoral immune response was initiated in the NSs-deletant virus-infected animals. OD signals were still detectable when fresh serially diluted serum samples were assayed at 58 d (approximately 8 wk) p.i. prior to the administration of a lethal virus challenge with CB1/2014.

To further characterize the immunogenicity of each recombinant virus, we evaluated in vitro neutralization capabilities of the ferret sera by using a 50% focus reduction neutralization test (FRNT_50_) with the serum samples collected at the indicated time points post initial virus challenge (Fig. 4 *D*–*F*). Similar to the ELISA data presented in Fig. 4 *A*–*C*, the parental rHB29-infected ferrets showed the highest neutralization titers against both genotype D (HB29 and CB8/2016) and B (CB1/2014) viruses. Neutralization titres recorded from the rHB29-infected animal sera were detectable by day 8 p.i. and peaked at day 14 p.i. The peak FRNT_50_ values obtained at day 14 p.i. were 5.5, 5.8, and 4.3 FRNT_50_ (10log_2_) for the neutralization of rHB29, CB8/2016, or CB1/2014 viruses, respectively (Fig. 4 *D*–*F*). Although the FRNT_50_ value of sera from rHB29NSsP_102_A- and rHB2912aaNSs-infected ferrets at 8 d p.i. was lower than the sera from that of the parental rHB29-infected group, they showed significantly increased FRNT_50_ values by 14 d p.i. from 2.5 to 3.3 FRNT_50_ (10log_2_) against genotype D strains (rHB29 and CB8/2016). Further, both sera from rHB29NSsP_102_A- and rHB2912aaNSs-infected animals showed neutralization titers of 2.5 FRNT_50_ (10log_2_) against heterologous genotype B SFTSV strain, CB1/2014 on day 14 p.i. (Fig. 4*F*). The FRNT_50_ values of each ferret sera remained stable until 58 d p.i. (Fig. 4 *D*–*F*). These results suggest that preimmunization with rHB29NSsP_102_A or rHB2912aaNSs could induce robust production of cross-neutralizing antibodies against homologous and heterologous SFTSV strains.

### Immunization of Animals with Recombinant SFTS Viruses Confers Cross-Clade Protection in a Lethal Challenge Model.

**Fig. 5.**
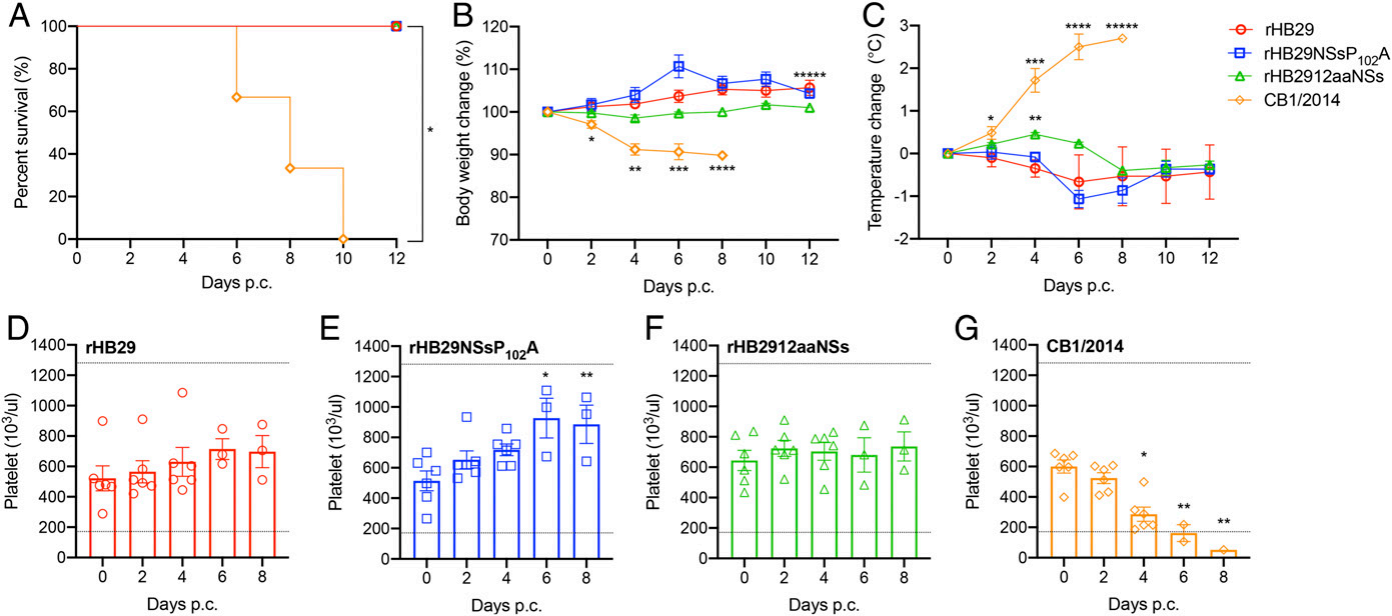
Survival, relative body weight, temperature change and hematological analysis of immunized ferrets in a lethal challenge model. Six ferrets in each group were i.m. inoculated with 10^7.6^ TCID50 of CB1/2014. Survival (*A*) relative body weight (*B*) and temperature (*C*) were assessed and are shown as SEM. Data (means ± SEM) are presented in *B* and *C*. Blood from infected ferrets was collected every other day and hematological examination was performed using a Celltac hematology analyzer. Platelet counts from ferrets preimmunized with rHB29 (*D*), rHB29NSsP_102_A (*E*), or rHB2912aaNSs (*F*) and subsequently challenged with CB1/2014 or animals infected with CB1/2014 only (*G*) are shown. CB1/2014 infected ferrets were additionally infected for positive control. Normal range of platelet counts in ferrets are 171.7 to 1,280.6 × 10^3^/µL (46, 58). The dashed lines indicate the normal value range for platelet counts. The asterisks indicate significance compared to each day p.i. sample. The 2-tailed Mantel–Cox method (*A*) or the one-way ANOVA with Dunnett multiple comparison test (*B*–*F*) was used to assess *P* values by the 2-tailed, unpaired *t* test. *P = 0.0025 (*A*); *P < 0.02, **P < 0.0001, ***P = 0.0053, ****P = 0.0019, and *****P = 0.0478 (*B*); *P = 0.0377, **P = 0.0140, ***P < 0.0001, ****P = 0.0025, and *****P = 0.0243 (*C*); *P = 0.0055 and **P = 0.0124 (*E*); and *P = 0.0003 and **P = 0.0005 (*G*).

We next wanted to examine whether a single prior infection of the ferrets with either rHB29, rHB29NSsP_102_A, or rHB2912aaNSs conferred protective immunity. At 8 wk p.i., the surviving animals were challenged with the genotype B, CB1/2014 strain of SFTSV. Six animals in each group were challenged i.m. with 5 × 10^7^ plaque-forming units (PFUs) and their survival, weight change, temperature, and viral RNA loads were measured for 12 d. At 5 d postchallenge (p.c.), 3 animals were killed per group to analyze tissue tropism and assess viral dissemination by qRT-PCR (Fig. 5). Remarkably, all of the animals previously infected with either rHB29, rHB29NSsP_102_A, or rHB2912aaNSs survived the lethal challenge with CB1/2014, whereas all challenge virus only controls died by 10 d p.i. (Fig. 5*A*). No weight loss was recorded over the course of the infection in preimmunized animals (Fig. 5*B*), and only a mild transient fever was detected the rHB2912aaNSs-immunized ferrets (Fig. 5*C*). Further, no thrombocytopenia was detected in the immunized and challenged animals at any point during the infection time course (Fig. 5 *D*–*G*). Platelet counts in the rHB29- or rHB29NSsP_102_A-immunized ferrets rose from a mean count of 518 × 10^3^/μL on day 0 to 792 × 10^3^/μL on day 8 following CB1/2014 challenge (Fig. 5 *D* and *E*), whereas platelet counts remained stable in the rHB2912aaNSs-immunized, CB1/2014-challenged ferrets throughout the course of infection (Fig. 5*F*). Platelet counts in the CB1/2014-infected challenge only group were reduced from day 4 p.c. and continued to decrease until the cessation of the experiment (Fig. 5*G*).

### No Virus Replication Evident in Immunized Animals following Challenge.

**Fig. 6.**
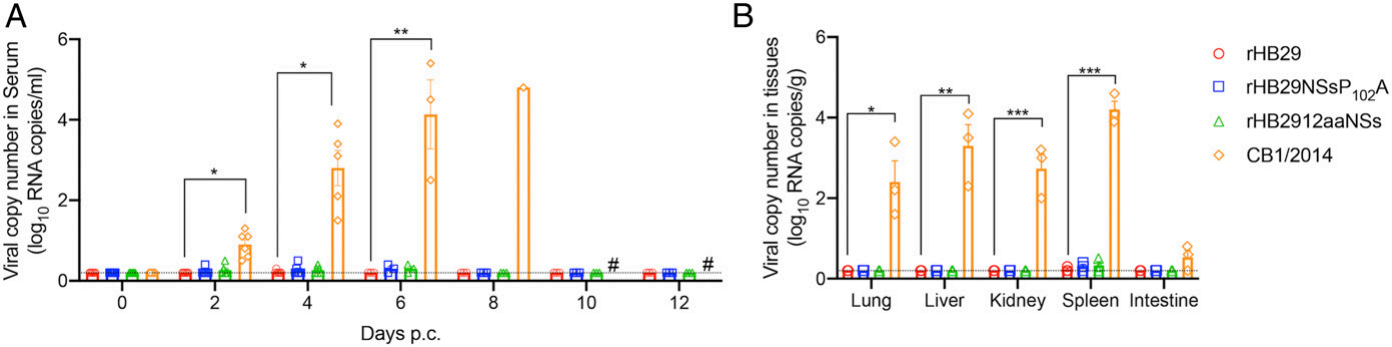
Distribution of viral RNA copies in blood and tissues of challenged ferrets. Six ferrets in each group were i.m. inoculated with 10^7.6^ TCID_50_ of CB1/2014. At the time points indicated following lethal challenge, serum viral RNA copy numbers were determined by real-time PCR (*A*). At 5 d p.i., 3 ferrets in each group were killed, and tissues (lung, liver, kidney, spleen, and intestine) were assessed for viral RNA copy number by real-time PCR (*B*). The one-way ANOVA with Dunnett multiple comparison test was used to assess *P* values compared with each day p.i. of rHB29. *P < 0.0001 and **P = 0.0005 (*A*); *P = 0.001, **P = 0.0001, and ***P < 0.0001 (*B*). # No samples were collected as animals had died/been killed.

Following the lethal challenge of immunized ferrets with CB1/2014, serum samples were analyzed for the presence of viral RNA (Fig. 6*A*). In rHB29NSsP_102_A-immunized ferrets, CB1/2014 viral RNA was detected in the serum at 0.27 log_10_ viral copies/mL at 2 d p.c., reaching peak copy numbers of 0.3 log_10_ viral copies/mL at day 6 p.c. A similar pattern of serum viral RNA copy numbers was recorded in CB1/2014-challenged rHB2912aaNSs-immunized animals, whereas no viral RNA was detected in the serum of the rHB29-immunized group. CB1/2014 viral RNA was found in the serum of the challenge only group and was detectable from day 2 p.c. (0.9 log_10_ viral copies/mL) and continued to rise until day 8 p.c. (4.8 log_10_ viral copies/mL; Fig. 6*A*). No changes in blood cell parameters were noted in either rHB29NSsP_102_A- or rHB2912aaNSs-immunized animals following virus challenge (*SI Appendix*, Fig. S2 *A* and *B*). Transient increases in serum ALT or AST levels were noted in rHB29- or rHB2912aaNSs-immunized animals after virus challenge (*SI Appendix*, Fig. S2 *C* and *D*), while both ALT and AST levels rose steadily over the course of infection in the blood samples taken from the CB1/2014 challenge only animals (*SI Appendix*, Fig. S2 *C* and *D*).

At 5 d p.c., 3 animals were killed per group to assess virus dissemination and tissue tropism in the challenged animals. In concordance with the initial infection data for CB8/2016 (Fig. 2*D*), the organ with the highest detectable viral RNA levels following challenge of the immunized animals was the spleen (Fig. 6*B*). Other than the viral RNA detected in the challenge control group, the greatest amount of viral RNA was detected in the spleens isolated from the rHB29NSsP_102_A-immunized and CB1/2014 challenged group, although this increase was not statistically significant (mean value of 0.3 log_10_ viral copies/g). When the other tissues (e.g., lung, liver, kidney, and intestine) were examined, no statistically significant increases in viral RNA levels were detected (Fig. 6*B*).

### Genetic Stability of rHB2912aaNSs.

To evaluate the genetic stability of the NSs-deletant virus (rHB2912aaNSs), we conducted 6 serial passages of rHB2912aaNSs virus through groups of ferrets. In detail, 2 ferrets were infected with rHB2912aaNSs and the spleens of the animals were collected at 4 d p.i. for virus isolation in Vero-E6 cells. Once isolated, the passaged virus was administered to another pair of animals, until 6 passages had been recorded. The viral S RNA segment was amplified from each animals’ splenic tissue by RT-PCR, and the presence of the NSs truncation was confirmed by sanger sequencing. There were no nucleotide substitutions detected in the sequence of the S RNA segment isolated from passaged viruses compared to the original virus stock. Sequencing results also showed that no revertant virus was detected after 6 serial passages through sequentially infected animals.

## Discussion

SFTSV is an emerging pathogen of global significance (9, 47). Since its emergence in China in 2009 (2), the virus has been isolated in Japan (3), Korea (4), and Vietnam (7). As SFTSV is a relatively novel disease, therapeutic options are still limited to the administration of ribavirin, steroids, and/or plasma exchange in human patients (48). Several companies and laboratories have been working to develop vaccines against SFTSV (43, 49). However, there are many hurdles that must be overcome to successfully produce a useable vaccine, including the establishment of reliable vaccine efficacy testing methods. Firstly, the creation of proper animal infection models will be necessary to evaluate vaccine efficacy in vivo. To date, only a few animal models, such as type I IFN-deficient (35), newborn (37), or mitomycin-treated mice (38), can recapitulate the fatal illness seen in human patients following SFTSV infection. However, it should be noted that type-I IFN-deficient and mitomycin-treated mouse models are both immunosuppressed systems. This means they cannot deliver the normal immune response against virus infection. Hence, a model for SFTSV infection with a normal immune status is essential to reliably test candidate vaccine efficacies. Secondly, a standard neutralization testing method will be needed to evaluate the cross-protective efficacy of novel vaccines. Recently, serum neutralizing antibody-based testing methods were reported (50); however, the cross-reactivity of these antibodies with different SFTSV genotypes still needs to be evaluated to define a standard strain to be used in vaccine production.

Here, we describe the evaluation of 2 live-attenuated vaccine candidates against SFTSV, based on the wild type-derived rHB29 strain. We demonstrate the immunogenicity of the viruses following infection and show prior immunization induces protection against a lethal SFTSV infection in a ferret challenge model. In our previous study, we demonstrate that the aged ferret could reproduce SFTS-like pathogenesis following infection and that the resulting disease fully recapitulates the clinical manifestations of human SFTSV infections. We also show IFN-mediated antiviral signaling is important for SFTSV pathogenesis through transcriptomic analysis. These data suggest that our immune-competent age-dependent ferret model will be useful for the development of anti-SFTSV therapies and vaccine development (40). Our results show that both NSs-mutant viruses (rHB29NSsP_102_A and rHB2912aaNSs) have reduced pathogenicity in the ferret and infected animals did not display any SFTS-like clinical disease in comparison to animals infected with the CB8/2016 strain (genotype D). It is noteworthy that virus RNA copy number in sera and target organs of rHB2912aaNSs-infected ferrets were significantly lower than those of the rHB29-infected ferrets. However, infected ferrets elicited a strong IgG and a neutralizing antibody response following a single immunization with rHB2912aaNSs. Further, rHB2912aaNSs was only detected from 4 to 6 d p.i. in the liver and spleens of infected animals, which are important immune defenses of the body. Therefore, we hypothesize that the low levels of viral RNA detected in the sera and tissues of rHB2912aaNSs-infected ferrets result in sufficient antigen presentation in the spleen or liver to induce a strong, protective, antibody response from a single dose of virus.

Safety is a major consideration for live-attenuated virus vaccines. Concerns regarding a reversion to virulence of the attenuated virus by genetic drift are often raised. However, as rHB2912aaNSs contains a 96% deletion of the NSs ORF rather than individual residue mutations, there is minimal chance of virus reversion to wild type through the acquisition of the deleted gene sequences during passage. When we conducted serial passaging of rHB2912aaNSs virus in vitro and in vivo, no revertant virus was detected during multiple sequential passage (up to 6 times in ferrets), demonstrating the genetic stability and safety of this vaccine candidate. For rHB29NSsP_102_A, we did not detect a revertant mutation in cell culture experiments. However, its genetic stability during in vivo passaging remains to be studied. Genetic segment reassortment of closely related viruses with our vaccine candidates remains theoretically possible and reassortment is well documented among the *Bunyavirales* and SFTS viruses (51). However, any genetic reassortment with rHB2912aaNSs would result in either an attenuated virus containing the rHB2912aaNSs-derived S RNA segment or the initial parental viruses.

Recently, genetic and phylogenetic analysis of SFTS viruses revealed that SFTSV could be grouped into at least 6 different genotypes referred to as genotypes A to F and that the prevalence of SFTSV genotypes are varied dependent on country (52). Since the recombinant SFTS viruses described herein belonged to genotype D, the immunized ferrets were subsequently infected with a known lethal dose of a heterologous genotype B strain (CB1/2014) to evaluate cross-protective efficacy. These data clearly demonstrate that a single immunization of rHB29NSsP_102_A or rHB2912aaNSs is sufficient to confer heterologous-genotype protection in an aged ferret model when challenged with a lethal dose of the virulent strain of SFTSV, CB1/2014. Further, this protective effect was evidenced by a lack of clinical manifestations, an absence of thrombocytopenia and reduced virus replication and dissemination to the surrounding tissues of infected animals.

Further research will need to be undertaken into the development of new reverse-genetics systems to fully understand the differences noted in the in vivo virulence observed between the Korean-derived isolates (CB1/2014 and CB8/2016) and the Chinese isolate (HB29) in our ferret model. The sequence of the recombinant rHB29 virus is based on a strain of SFTSV isolated from a patient described in Yu et al. (2). As shown in Table 1, there are several differences between the viral isolates at the amino acid level in key proteins such as the virulence factor and innate immune antagonist NSs. The NSs protein of HB29 is only 96.6 and 98.0% similar to the NSs of CB1/2014 or CB8/2016, respectively. Our own experimental data has demonstrated a single point mutation (such as P_102_A) within NSs can dramatically reduce virulence of a virus (31). There is a total of 29 or 50 amino acid substitutions between HB29 and CB8/2016 or CB1/2014, respectively (*SI Appendix*, Table S1). Therefore, it is possible that any of the amino acid changes between the different isolates could result in the attenuated phenotype observed in HB29-infected ferrets. Future work should aim to identify and pinpoint the amino acid changes that increase the pathogenicity of the Korean isolates.

**Table 1.** Comparison of percentage amino acid sequence identities of strains used in this study to the published sequence of HB29 (AJD86038 [L], AJD86039 [M], AJD86041 [NSs], AJD86040 [N])

Genotype	Strain	Amino acid sequence identity, %			
		L (RdRp)	M (Gn/Gc)	S (N)	S (NSs)
Genotype B	CB1/2014	99.1	97.7	98.8	96.6
Genotype D	CB8/2016	99.4	98.5	99.6	98.0

In conclusion, we evaluated 2 recombinant HB29 derived live-attenuated SFTSV vaccine candidates and demonstrated their immunogenicity and protective efficacy using a lethal model for SFTSV in ferrets. rHB29NSsP_102_A or rHB2912aaNSs virus infection showed significantly reduced pathogenicity in the aged ferrets compared with the homologous genotype CB8/2016 strain (genotype D). Both viruses induced strong IgG and neutralizing antibody responses against homologous and heterologous SFTSV strains. In particular, we found that following a single immunization with rHB29NSsP_102_A or rHB2912aaNSs ferrets were completely protected from lethal SFTSV challenge. No virus RNA was detectable in the serum or organs of preimmunized animals that had been challenged with CB1/2014 (Fig. 6), demonstrating the induction of sterilizing immunity. This suggests that a live attenuated vaccine platform might represent a suitable vaccine strategy against SFTSV due to its ability to induce a wider range of immune response types. It was recently reported that a live attenuated rVSV-based vaccine expressing the SFTSV Gn/Gc glycoproteins can elicit protection against SFTSV in immunocompromised IFNAR^−/−^ mice (43); however, our current study represents a substantial contribution toward the development of an effective preventive vaccine for SFTS. Our live attenuated vaccines induced complete protection against lethal SFTSV challenge in an immunocompetent middle-sized animal model that exhibits clinical manifestations seen in SFTS patients (40). Given the genetic diversity of SFTSV in nature, few prior studies have examined the cross-protective immunity against heterologous SFTSV genotype infections; thus, our present study provides valuable insights into the design of preventive vaccines against SFTSV.

## Methods

### Cells and Viruses.

Vero E6 cell line (commonly used cell line originally obtained from European Collection of Authenticated Cell Cultures, previously described in ref. 53) was grown in Dulbecco's modified Eagle’s medium (DMEM) supplemented with 10% fetal calf serum (FCS). HuH7-Lunet-T7 cells (54), which stably express T7 RNA polymerase, were obtained from R. Bartenschlager and were grown in DMEM supplemented with 2 mM l-glutamine, nonessential amino acids, and 10% FCS. All cell lines were grown at 37 °C with 5% CO_2_ unless otherwise stated.

SFTSV strains used in this study were a recombinant derivative of the Hubei-29 strain generated by reverse genetics (rHB29). This sequence is based on a plaque-purified stock called Hubei 29pp (HB29pp) provided by Amy Lambert (CDC Arbovirus Diseases Branch, Division of Vector-Borne Infectious Diseases, Fort Collins, CO) (55). CB1/2014 and CB8/2016 were isolated from the sera of SFTSV-infected patients hospitalized with SFTS symptoms at Chungbuk National University Hospital, South Korea (45). Stock viruses were generated by propagation of the virus up to a maximum of 5 times from the original patient sera.

Working stocks of SFTSV were generated in a Vero E6 cell line by infecting at a low multiplicity of infection and harvesting the cell culture medium 7 d p.i. In vitro experiments with SFTSV conducted in the United Kingdom were performed under containment level 3 conditions, approved by the UK Health and Safety Executive. In Korea, viruses were handled in an enhanced biosafety level 3 containment laboratory as approved by the Korean Centres for Disease Control and Prevention (KCDC-14-3-07).

### Plasmids.

Plasmids for the recovery of SFTSV have been described previously (55). pTM1-HB29ppL and pTM1-HB29N contain the SFTSV HB29 L and N ORFs under the control of T7 promoter and encephalomyocarditis virus internal ribosome entry site sequence; pTVT7-HB29S, pTVT7- HB29M, and pTVT7-HB29ppL contain full-length complementary DNAs to the SFTSV strain HB29 antigenome segments flanked by T7 promoter and hepatitis delta ribozyme sequences. Plasmid pTVT7-HB29S12aaNSs, which was previously described (33), contains an internal deletion in the coding region of NSs (Δ2-282), leaving the methionine and last 11 amino acids of the NSs ORF intact. The plasmid was used in the recovery of rHB2912aaNSs. Plasmids used to generate rHB29NSsP_102_A were described in previous work (31). All cDNA constructs were confirmed by Sanger sequencing. Recombinant viruses were recovered from cDNA plasmid transfection as previously described (33, 55).

### Virus Titration by Plaque- or Focus- Forming Assays.

Virus titres were determined by focus-forming assays or by plaque assay in Vero E6 cells. Briefly, confluent monolayers of Vero E6 cells were infected with serial dilutions of virus made in phosphate-buffered saline (PBS) containing 2% FCS and incubated for 1 h at 37 °C, followed by the addition of a Glasgow’s Minimum Essential Medium overlay supplemented with 2% FCS and 0.6% Avicel (FMC Biopolymer). The cells were incubated for 6 d before fixation and staining with crystal violet to visualize SFTSV plaques, or using focus-forming assays for recombinant viruses as described previously (33, 55).

### Infection of Ferrets with Recombinant Viruses.

Groups of aged ferrets (>4 y old; *n* = 12) were infected via the i.m. route with rHB29 (4 × 10^6^ PFUs), rHB29NSsP102A (4 × 10^6^ PFUs), rHB2912aaNSs (5 × 10^5^ PFUs), and CB8/2016 (5 × 10^5^ PFUs). Blood was collected from anesthetized ferrets at intervals of 2 d until 12 d p.i. for virus titrations. In addition, 500-μL blood samples were immediately stored in ethylenediaminetetraacetic acid (EDTA) tubes (MEDISTAR, Seoul, Korea) and analyzed for hematological parameters using a Celltac hematology analyzer (MEK-6550J/K; Nihon Kohden). To assess virus replication in SFTSV-infected ferrets, 3 ferrets from each group were scarified at 4 and 6 d p.i., and tissues (lung, liver, spleen, kidney, and intestine) were collected to assess viral RNA loads.

### Quantification of Viral Copy Numbers by qRT-PCR.

Total RNA from infected ferret sera and organ tissues was extracted using TRIzol LS Reagent (Thermo Fisher Scientific), and cDNA was generated by reverse transcription with specific (SFTSV-S) and random (Takara) primers using the M-MLV Reverse Transcriptase (Enzynomics). Viral copy numbers were determined by quantitative real-time RT-PCR using an S segment-based SFTSV-specific primer set: forward primer (SFTSV-S-F), gcagttggaatcaggga; and reverse primer (SFTSV-S-R), cccacttggacatgtgct. Copy numbers were calculated as a ratio with respect to the standard control as previously described (36), and real-time PCR reactions were performed using a SYBR Green Supermix (Bio-Rad) and a CFX96 Touch Real-Time PCR Detection System (Bio-Rad). The limit of detection of qRT-PCR was 2 RNA genome copies per reaction.

### SFTSV Antibodies Detected by ELISA and Virus Neutralization Assay.

For the SFTSV ELISA, plates were coated overnight at 4 °C with purified nucleocapsid protein (0.2 μg/well) of CB1/2014 strain as previously described (56). Ferret sera was heat inactivated and treated with kaolin to reduce nonspecific binding. For the ELISA experiment, serum was collected from CB1/2014 infected ferrets and was used as a positive control in the data presented. Briefly, serum samples were diluted 1:10, followed by 2-fold dilution series in PBS from 1:10 to 1:2,560. After washing, the plates were incubated with 100 μL of diluted ferret serum with 2% skim milk in 0.05% PBS-Tween 20 for 2 h at room temperature. Plates were then incubated with horse radish peroxidase (HRP)–anti-ferret IgG (KPL). For detection of antibodies, the plates were overlaid with *O*-phenylenediamine dihydrochloride (Sigma) substrate and 1 M sulfuric acid was added. OD was measured with a spectrometer (iMark Microplate Reader; Bio-Rad) at 450 nm.

Ferret sera were collected at 0, 8, 14, and 58 d after the last inoculation and neutralizing antibody was measured by FRNT_50_ as previously described (57). Briefly, heat-inactivated, 1:10-diluted sera were used to neutralize virus. Twenty-five microliters of serially diluted serum were mixed with an equal volume of 200 focus-forming units of each SFTSV strain (rHB29, CB8/2016, and CB1/2014) for 1 h 37 °C. The mixture was adsorbed on to Vero E6 cells in 96-well plate at 37 °C for 1 h. Cells were overlaid with maintenance medium, and the plates were incubated 37 °C in 5% CO_2_ for 5 d. The infected cells then were fixed with 10% formalin, and the foci formation was visualized with an in house-generated mouse anti-N antibody, followed by a HRP-conjugated anti-mouse IgG secondary antibody (The Jackson Laboratory). The antibody titers were expressed as reciprocals of the highest serum dilution showing a 50% plaque reduction or greater compared with control values.

### Lethal Challenge of SFTSV in Ferrets.

Ferrets immunized with recombinant viruses or PBS-preimmunized control animals were challenged i.m. with 10^7.6^ TCID_50_ of CB1/2014 strain (0.5 mL in the outside of each thigh of both legs). Throughout the ferret challenge studies, we used a TCID_50_ dose (∼10^7.6^) of SFTSV. This dose is known to cause 100% fatality rate in infected ferrets, as seen in our recent study (40). Survival was monitored for 14 d after lethal SFTSV challenge with CB1/2014. Sera were collected from each ferret at 2-d intervals after infection and peripheral virus RNA copy numbers were determined. Hematological parameters were analyzed using EDTA-treated whole-blood samples from infected animals using the Celltac hematology analyzer (MEK-6550J/K; Nihon Kohden). Biochemical parameters of serum from infected animals were determined using Celltac α (MEK-6550; Nihon Kohden).

### Ethics Statement.

All animal experiments were approved by the Medical Research Institute, a member of Laboratory Animal Research Centre of Chungbuk National University (approval no. CBNUA-1234-18-01) and were conducted in strict accordance and adherence to relevant policies regarding animal handling as mandated under the Guidelines for Animal Use and Care of the Korea Centre for Disease Control.

### Data Availability.

The raw data that underpin the findings of this study are openly available from Enlighten Research Data at https://dx.doi.org/10.5525/gla.researchdata.897. Authors will make reagents described in this study available on request (by qualified researchers for their own use). Requests should be directed to the corresponding authors Y.K.C. or B.B.

## Supplementary Material

Supplementary File
