# Supplementary material for: Cross-genotype protection of live-attenuated vaccine candidate for severe fever with thrombocytopenia syndrome virus in a ferret model
Source: Proc Natl Acad Sci U S A. 2019 Dec 9;116(52):26900–8. doi: 10.1073/pnas.1914704116 (PMC6936527; doi:10.1073/pnas.1914704116)
Supplement: Supplementary File [file pnas.1914704116.sapp.pdf]

PNAS
www.pnas.org

Supplementary Information for

Cross-genotype protection of live-attenuated vaccine candidate for severe fever with thrombocytopenia syndrome virus in a ferret model.

Kwang-Min Yu[1], Su-Jin Park[1], Min-Ah Yu[1], Young-Il Kim[1], Younho Choi[2], Jae U. Jung[2], Benjamin Brennan[3]* & Young Ki Choi[1]*

Young Ki Choi
Email: choiki55@chungbuk.ac.kr

Benjamin Brennan
Email: ben.brennan@glasgow.ac.uk

This PDF file includes:

Figures S1 to S2
Tables S1

www.pnas.org/cgi/doi/10.1073/pnas.1914704116

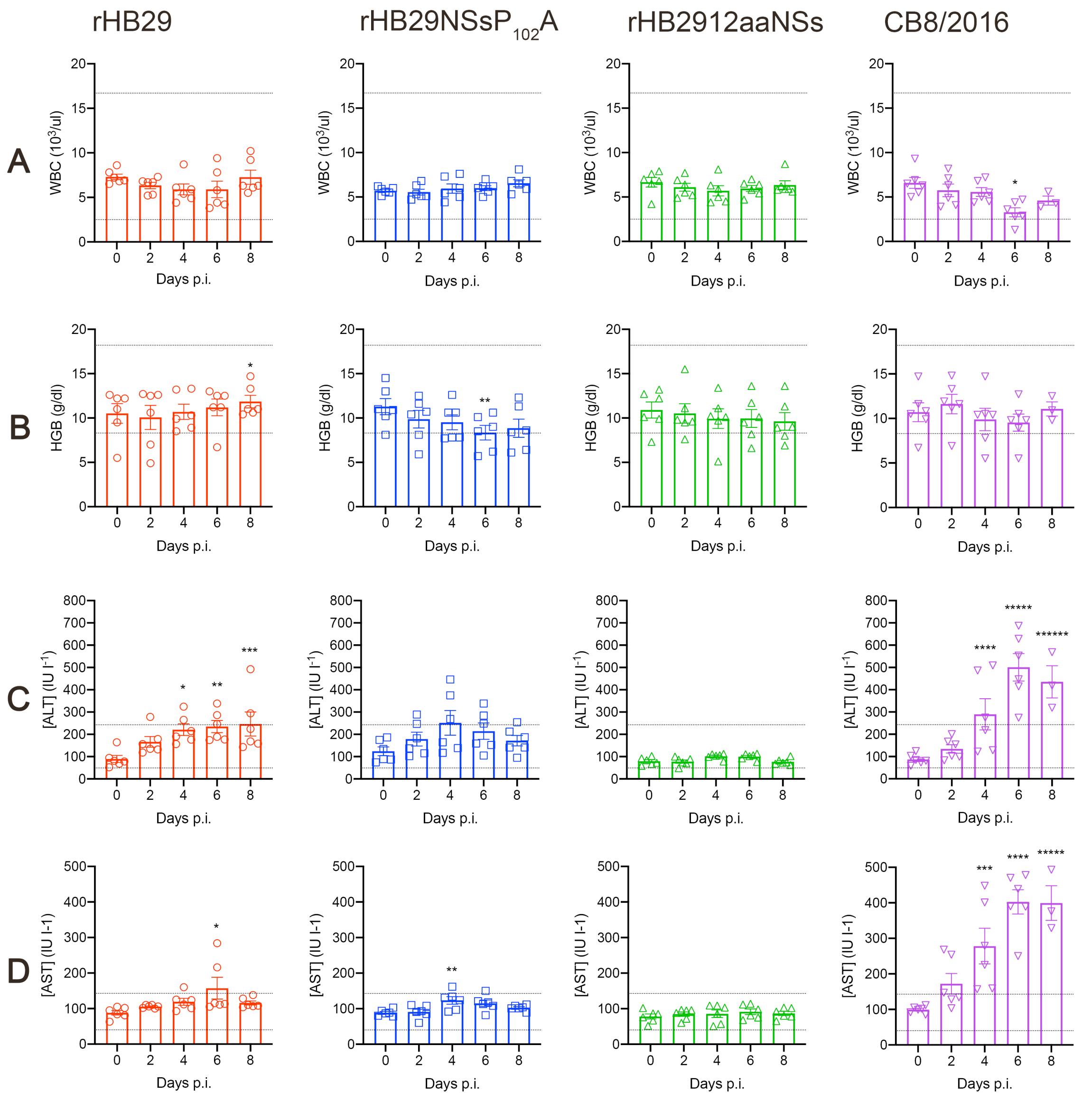

Fig S1. Hematological analysis of aged ferrets infected with recombinant or CB8/2016 SFTS viruses. (A-D) The aged ferrets in each group were inoculated with 4 $x10^6$ PFU of rHB29 (red), 4 $x10^6$ PFU of $rHB29NSsP_{102}A$ (blue), 5 $x10^5$ PFU of rHB2912aaNSs (green) or 5 $x10^5$ PFU of CB8/2016 (purple) and White blood cell, WBC **(A)**, Haemoglobin, HGB **(B)**, Alanine transaminase, ALT **(C)** and Aspartate transaminase, AST **(D)** were analyzed until 8 days p.i. (n=6). The mean values are shown ± standard error the mean. The asterisks indicate significance compared to each day p.i. sample by the one-way ANOVA with Dunnett's multiple comparison test. *P =0.0017 **(A)**; *P = 0.0265, **P = 0.0129, ***P = 0.007, ****P = 0.0244, *****P < 0.0001, and ******P = 0.0015, **(C)**; and *P = 0.0107, **P = 0.0092, ***P = 0.004, ****P < 0.0001, and *****P= 0.0002 **(D)**.

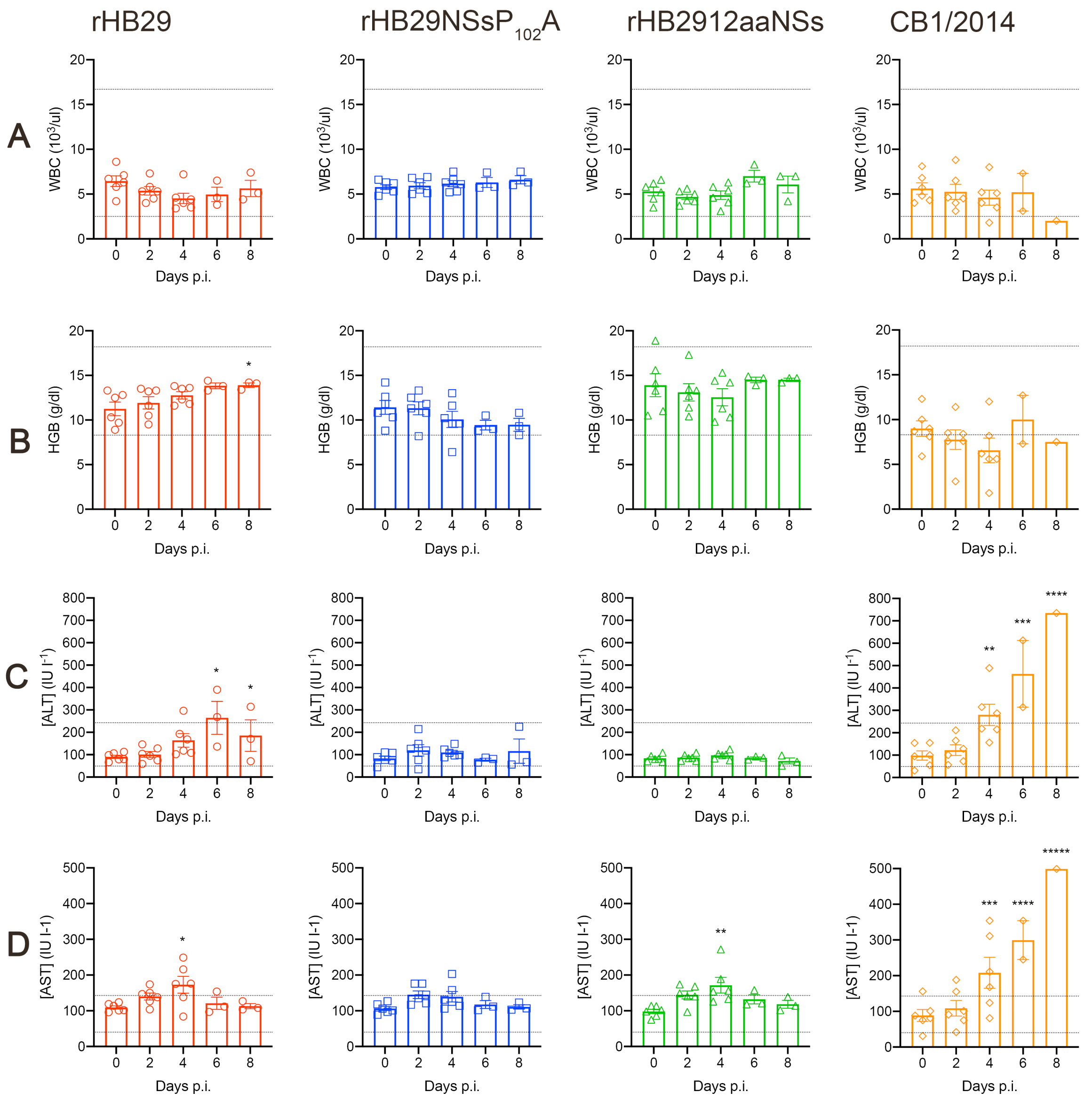

Fig S2. Hematological analysis of aged ferrets after challenged with CB1/2014 SFTS viruses. (A-D) Groups of ferrets vaccinated with rHB29 (red), rHB29NSsP$_{102}$A (blue), rHB2912aaNSs (green), respectively was challenged withCB1/2014. PBS-immunised ferrets were also infected with CB1/2014 (orange) as a control. WBC **(A)**, HGB **(B)**, ALT **(C)** and AST **(D)** were analyzed until 8 days p.i. (n=6). The mean values are shown ± standard error the mean. The asterisks indicate significance compared to each day p.i. sample by the one-way ANOVA with Dunnett's multiple comparison test. *P =0.0493 **(B)**; *P = 0.0095, **P = 0.0154, ***P = 0.0009, and ****P < 0.0001 **(C)**; and *P = 0.0160, **P = 0.0493, ***P = 0.0042, ****P = 0.0423, *****P = 0.0101, and ******P = 0.0003 **(D)**.

Table S1. Identity of amino acid differences in open reading frames of SFTSV genotypes B and D. L (RNA-dependent RNA polymerase), M (M polyprotein), NSs (non-structural protein) and N (nucleocapsid protein) open reading frames.

		Amino acid in			
		Genotype D	Genotype D	Genotype D	Genotype B
Gene	Position	HB29*	CB8/2016[#]	NSsP$_{102}$A*	CB1/2014[¶]
L	2	N	D	N	D
	264	D	D	D	N
	314	G	G	G	S
	566	F	Y	F	Y
	651	V	I	V	I
	719	N	S	N	S
	795	S	F	S	S
	835	K	R	K	R
	856	K	K	K	R
	1038	T	S	T	S
	1397	S	N	S	N
	1433	A	T	A	A
	1447	M	M	M	T
	1684	K	R	K	R
	1717	V	V	V	I
	1745	S	S	S	N
	1903	N	N	N	S
M	21	S	S	S	T
	37	G	G	G	N
	114	E	G	E	G
	218	G	G	G	S

	272	K	K	K	E
	273	A	A	A	T
	300	E	D	E	E
	321	T	M	T	M
	371	R	K	R	K
	385	S	T	S	T
	3944	H	Q	H	Q
	404	T	T	T	A
	457	L	I	L	L
	470	T	T	T	S
	501	V	M	V	M
	506	G	G	G	R
	525	I	V	I	V
	587	P	P	P	S
	662	T	T	T	A
	737	I	T	I	T
	960	R	S	R	S
	962	D	G	D	D
	995	T	T	T	S
	1011	N	N	N	S
	1017	F	S	F	S
	1058	L	S	L	S
	1070	T	T	T	S
NSs	102	P	P	A	P
	130	R	K	R	R
	197	L	M	L	M
	207	P	S	P	S

	223	V	V	V	I
	228	T	T	T	A
	243	V	A	V	V
	245	H	Q	H	Q
	249	H	H	H	Y
	278	I	I	I	V
	281	R	R	R	K
	291	K	K	K	E
N	51	V	V	V	M
	52	K	K	K	E

* based on accession numbers AJD86038 (L), AJD86039 (M), AJD86041 (NSs), AJD86040 (N).
[#] based on accession numbers QDF82697 (L), QDF82698 (M), QDF82699 (NSs), QDF82700 (N).
[¶] based on accession numbers ASW22982 (L), ASW22985 (M), ASW22988 (NSs), ASW22989 (N).
